# Non-catalytic hydrogenation of VO_2_ in acid solution

**DOI:** 10.1038/s41467-018-03292-y

**Published:** 2018-02-26

**Authors:** Yuliang Chen, Zhaowu Wang, Shi Chen, Hui Ren, Liangxin Wang, Guobin Zhang, Yalin Lu, Jun Jiang, Chongwen Zou, Yi Luo

**Affiliations:** 1 National Synchrotron Radiation Laboratory, University of Science and Technology of China, Hefei, 230029 China; 2 Hefei National Laboratory for Physical Sciences at the Microscale, Collaborative Innovation Center of Chemistry for Energy Materials, CAS Center for Excellence in Nanoscience, School of Chemistry and Materials Science, University of Science and Technology of China, Hefei, 230026 China; 3 School of Physics and Engineering, Henan University of Science and Technology, Henan Key Laboratory of Photoelectric Energy Storage Materials and Applications, Luoyang, 471023 Henan China

## Abstract

Hydrogenation is an effective way to tune the property of metal oxides. It can conventionally be performed by doping hydrogen into solid materials with noble-metal catalysis, high-temperature/pressure annealing treatment, or high-energy proton implantation in vacuum condition. Acid solution naturally provides a rich proton source, but it should cause corrosion rather than hydrogenation to metal oxides. Here we report a facile approach to hydrogenate monoclinic vanadium dioxide (VO_2_) in acid solution at ambient condition by placing a small piece of low workfunction metal (Al, Cu, Ag, Zn, or Fe) on VO_2_ surface. It is found that the attachment of a tiny metal particle (~1.0 mm) can lead to the complete hydrogenation of an entire wafer-size VO_2_ (>2 inch). Moreover, with the right choice of the metal a two-step insulator–metal–insulator phase modulation can even be achieved. An electron–proton co-doping mechanism has been proposed and verified by the first-principles calculations.

## Abstract (web summary)

Hydrogenation is an effective way to tune the property of metal oxides. Here, the authors report a simple approach to hydrogenate VO_2_ in acid solution under ambient conditions by placing a small piece of low workfunction metal on VO_2_ surface.

## Introduction

As a typical transition oxide, VO_2_ has a pronounced metal–insulator transition (MIT) behavior at the critical temperature near 68 °C, accompanying by a sharp resistance change up to five orders of magnitude and marked infrared switching effect within sub-*ps* time scale^1–5^. It has thus shown great potential for important applications in memory material^6,7^, smart window^8,9^ and ultra-fast optical switching device^10^. Many efforts have been devoted to improve the phase transition properties of metal oxides^11–16^, including the hydrogenation treatment, which has been demonstrated to be an effective way to tune the property of metal oxides^17–21^. Recent experiments observed that H-incorporations in M-VO_2_ could result in a very stable metallic phase at room temperature^19,22^, giving excellent thermoelectric performance^23^. Moreover, further injecting H into the lightly doped M-VO_2_ could create another insulating state at the heavily H-doping situation^20^, enabling the control of MIT in a reversible and consecutive manner. Previous studies examined the thermodynamic and kinetic properties of H or Li doping in VO_2_ lattice^22,24,25^, showing that H atoms preferred to diffuse along the *c*-axis of rutile VO_2_ or *a*-axis of monoclinic VO_2_. Although the hydrogenation techniques available are not sustainable as they are conventionally performed with noble-metal (Au, Pt, Pd) catalysts, high-temperature/pressure annealing treatment or high-energy proton implantation in vacuum condition^22,26–28^.

In this work, we report a facile approach to hydrogenate monoclinic VO_2_ film in acid solution at ambient condition by placing a low workfunction metal particle (Al, Cu, Ag, Zn, or Fe) on VO_2_ surface. The workfunction difference will cause electron flowing from metal particles to VO_2_, which in turn drives surrounding solution protons to penetrate into VO_2_ due to electrostatic attraction, resulting a stable H atoms doping. This process will not only stabilize the VO_2_ lattice in acid, but also induce the modulation of phase transitions under ambience conditions, which should be of great potentials for material applications. An electron–proton co-doping mechanism has been proposed and this synergetic doping method will stimulate more simple and cost-effective atomic doping techniques in the future.

## Results

### Metal-acid treatment induced H-doped VO_2_ film

**Fig. 1:**
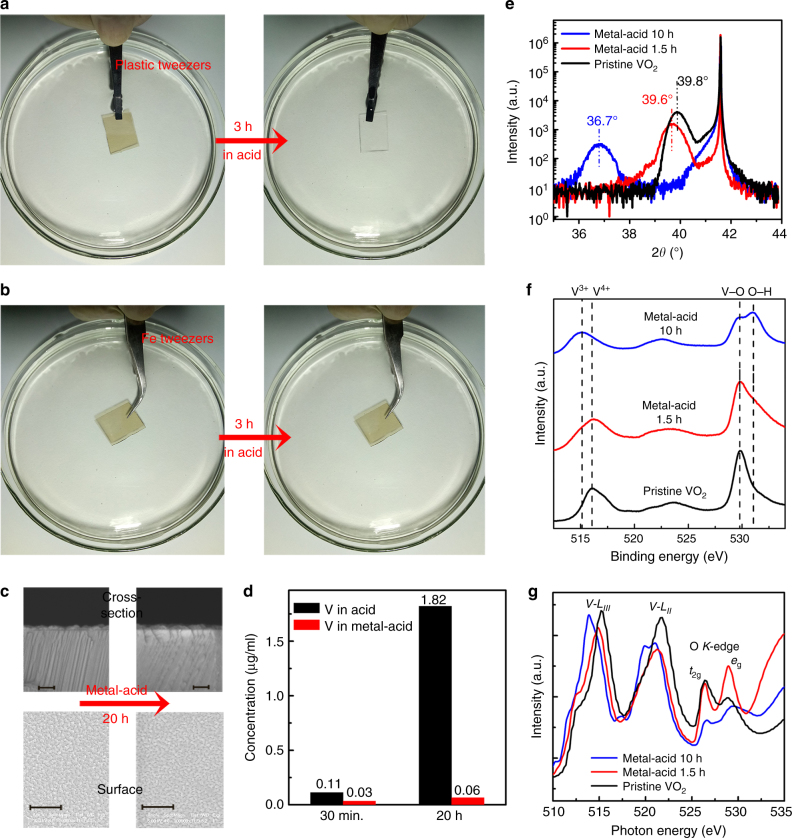
Metal-acid treatment induced hydrogenation of VO_2_ film. **a** The VO_2_ film on Al_2_O_3_ substrate held by a plastic tweezers was dissolved by 2%wt H_2_SO_4_ acid in 3 h. **b** Although a steel (Fe) tweezers attachment made the film intact in acid, showing pronounced anti-corrosion ability. **c** The SEM images for the cross-sections and surface morphologies of the VO_2_ film being treated by metal (Fe)-acid for 20 h, showing that the VO_2_ film maintains unchanged thickness and surface morphologies. The scale bar is 100 nm for the cross-sections and 500 nm for the surface morphologies, respectively. **d** Trace element analysis shows the V^4+^ concentrations in solution changing from 0.11 to 1.82 μg/ml after 30 min to 20 h with acid treatment, whereas very low V^4+^ concentration at 0.03–0.06 μg/ml is found at the same time period with metal (Fe)-acid treatment. The **e** XRD, **f** XPS, and **g** XANES characterizations for the pristine VO_2_ and metal (Fe)-acid-treated samples for 1.5 and 10 h, respectively. The pronounced (020) XRD peak shift from 39.8° to 36.7°, the increased V^3+^ and O–H XPS signals, and enhanced *e*_g_/*t*_2g_ XANES signal ratio (reflecting the variation of electron occupancy) along with the increase of metal (Fe)-acid time, indicate the lattice changes and O–H bonds formations due to light and heavy hydrogenations

Is it possible to use acid solution as a natural proton source to achieve the hydrogenation of VO_2_ at ambient condition? At the first glance, this appears to be an impossible mission, as the textbook tells us that pristine metallic oxides including VO_2_ are easily dissolved in acid through the well-established reaction of VO_2_ + 4H^+^ → V^4+^ + 2H_2_O. Indeed, as shown in Fig. 1a, when a 30 nm M-VO_2_/Al_2_O_3_ (0001) epitaxial film grown by molecular beam epitaxy method^29^ (Supplementary Fig. 1) held by a plastic tweezers was put into a 2%wt H_2_SO_4_ acid solution, the yellowy VO_2_ epitaxial film completely disappeared within 3 h. Although when a steel tweezers was used to hold the sample, as shown in Fig. 1b, the same VO_2_ film suddenly demonstrated excellent anti-corrosion ability: it remains intact after 3 h in the acid solution. Scanning electron microscope (SEM) images in Fig. 1c show that the thickness and morphology of VO_2_ film hardly change even after 20 h in acid solution. In addition, the atomic force microscope (AFM) measurements show nearly zero thickness variation for metal-acid-treated samples (Supplementary Fig. 2a), which is consistent with the SEM cross-section image and confirms the anti-corrosion ability. More convincingly, the trace element analysis in Fig. 1d revealed that the concentration of V^4+^ cations in solution increased from 0.11 to 1.82 μg/ml after immersing a VO_2_ film held by a plastic tweezers in acid from 30 min to 20 h, whereas it kept very low value at 0.03–0.06 μg/ml with a steel tweezers. All these results firmly point to the fact that the attachment of a metal can give excellent anti-corrosion ability to VO_2_.

Interestingly, the hydrogenated VO_2_ produced by Au or Pd catalyst is found to be very stable in acid solution (Supplementary Fig. 2b). One can thus reasonably assume that the anti-corrosion ability of such metal-acid-treated VO_2_ is due to the hydrogenation. The X-ray diffraction (XRD) spectra in Fig. 1e show the dynamic shifts of (020) diffraction peak from 39.8° to 36.7° after the metal-acid treatment due to the cell expansion caused by H-incorporation, which agree well with the results for lightly and heavily hydrogenated VO_2_ through conventional noble-metal catalysis at high temperature (Supplementary Fig. 3a). These hydrogenated VO_2_ films show successive metallic and insulator states as the hydrogen doping concentration increasing^30^.

The X-ray photoelectron spectroscopy (XPS) measurements presented in Fig. 1f clearly indicate the conversion from V^4+^ to V^3+^ state as the result of H intercalation, which is further confirmed by the variations of O1*s* peak at ~531.6 eV for the O–H species. The change of valence state from V^4+^ to V^(4−^*δ*^)+^ or even to V^3+^ state is also verified by the X-ray absorption near-edge structure (XANES) spectra in Fig. 1g as the V *L*-edge curves shift continuously to lower energy. After the metal-acid treatment, the relative intensity ratio of the *t*_2g_ and *e*_g_ peaks in O *K*-edge curves decreased substantially, reflecting the variation of electron occupancy due to electron doping as well as the loss of the *d*_//_ state upon hydrogenation^31^. All these spectroscopic features induced by metal-acid treatment for 1.5 and 10 h agree well with corresponding measurements on lightly and heavily hydrogenated VO_2_ through conventional catalysis techniques (Supplementary Fig. 3), respectively. It can thus be concluded that the metal-acid treatment can indeed create H-doping in the VO_2_ film.

### Hydrogenation of a wafer-size VO_2_ film

**Fig. 2:**
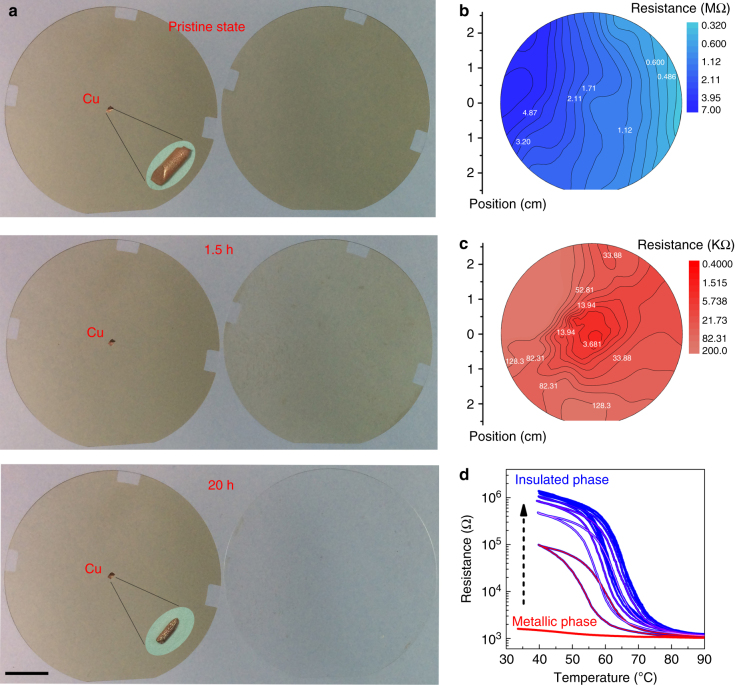
Hydrogenation of a wafer-size VO_2_ film with a tiny Cu particle. **a** The two 2-inch VO_2_/Al_2_O_3_ wafers immersed in 2%wt H_2_SO_4_ acid solution. The sample with a tiny copper (Cu) particle (~1 mm) attached on the center surface exhibits pronounced anti-corrosion ability, whereas the bare VO_2_/Al_2_O_3_ film is completely corroded within 1.5–3 h, leaving the transparent Al_2_O_3_ substrate. The scale bar is 1 cm. **b** The resistance mapping for the 2-inch pristine VO_2_ film. **c** The resistance mapping for the metal(Cu)-acid-treated VO_2_. For the whole 2-inch wafer, the surface resistance is decreased by almost three orders of magnitude in comparison to the pristine film, reflecting the MIT of M-VO_2_ by hydrogenation. **d** The resistance measurement in air for the metal(Cu)-acid-treated M-VO_2_ as the function of heated temperature. Along with the pronounced hysteresis *R*–*T* curve, the metallic sample gradually recovers to the initial insulated M-phase VO_2_

It is noted that contact area between the metal tweezers and the VO_2_ film is actually quite small. To further quantitatively explore the effect of metal attachment, we have placed a tiny Cu particle (~1.0 mm in diameter) at the center of one 2-inch M-VO_2_/Al_2_O_3_(0001) epitaxial film, and immersed them together into 2%wt H_2_SO_4_ solution. It is observed in Fig. 2a that the bare VO_2_ film with yellowy color could be dissolved within 1.5–3 h. In sharp contrast, the small copper particle has provided the protection for the whole 2-inch wafer from acid corrosion. In addition, when the Cu particle is taken away after the treatment, the film remains stable in acid solution as it has already been hydrogenated (Supplementary Fig. 4).

A key evidence about the hydrogenation of VO_2_ is the insulator–metal transition at room temperature^19,22^, i.e., the hydrogenation converts the insulated M-VO_2_ to the metallic phase (Supplementary Fig. 5). In comparison with the original insulating M-VO_2_ film (Fig. 2b), the surface resistance of above Cu-acid-treated sample is decreased by almost three orders of magnitude (Fig. 2c). After cyclically heating the sample in air between 40 and 90 °C for about 2 h, the intercalated hydrogens could be completely removed, and the film is recovered back to the insulated phase (Fig. 2d), which is consistent with the results obtained from hydrogenated samples through conventional catalysis (Supplementary Fig. 6). From Fig. 2a, one can note that the small copper particle can be taken away to leave out pure H-doping material. It is certainly a much better approach than the conventional catalysis-based technique as those metal catalysts (Au or Pt) sputtered onto the VO_2_ film surface are hardly removable. In addition, the latter gives only limited hydrogenation area covered by catalysts (Supplementary Fig. 5b).

### Hydrogenation effects controlled by different metals

**Fig. 3:**
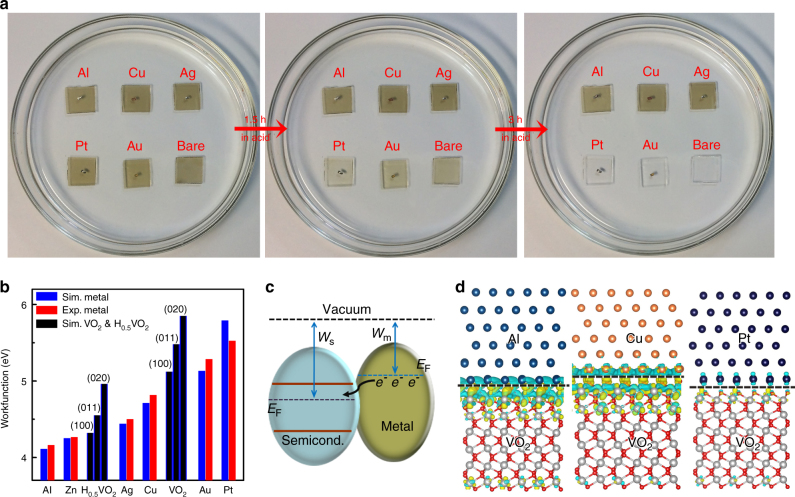
Hydrogenation effects induced by different metals. **a** Metals, such as Al, Cu, Ag, can protect M-VO_2_ (1 cm × 1 cm size) from corrosion in 2%wt H_2_SO_4_ acid solution, whereas metals, Pt and Au, can not. **b** Computed and experimental workfunction (*W*_F_) values for metals, VO_2_, and lightly hydrogenated H_0.5_VO_2_, with the order of Pt > Au > VO_2_ > Cu > Ag > H_0.5_VO_2_ > Zn > Al. **c** Schematic depiction of electrons flowing from metal with a higher Fermi level (i.e., lower workfunction *W*_m_) to semiconductor with a lower Fermi level (i.e., higher workfunction *W*_s_) at the interface. **d** Computed differential charge distribution at Al/Cu/Pt–VO_2_(020) interfaces, showing that active metals (Al and Cu) donate effective electrons to VO_2_. Green and yellow bubbles represent hole and electron charges, respectively. Gray, red, cyan, brown, navy beads stand for V, O, Al, Cu, Pt atoms, respectively

We have found that several other metals, such as Al, Cu, Ag, Zn, or Fe, can all induce hydrogenation and thereby protect VO_2_ from corrosion in acid, whereas Au and Pt can not. The effects of different metals are illustrated in Fig. 3a. One important parameter associated with the choice of the metal is the workfunction. The workfunction values calculated for the metals (Supplementary Fig. 7), VO_2_ and H_0.5_VO_2_ are plotted together with the reported experimental values^32^ in Fig. 3b. Simulations of pristine and hydrogenated VO_2_ systems were based on the most stable atomic models obtained by previous studies^30^. Because of high lattice symmetry, the electronic structure of H-doped VO_2_ is sensitive to the H-doping concentration but not to the atomic sites of H in lattice. By testing all of the 16 possible H-doping sites (Supplementary Fig. 8; Supplementary Table 1), we have taken the one with lowest energy for further investigation. A clear pattern can be observed: with the respect to the workfunction of VO_2_, the metal with smaller workfunction value can induce the hydrogenation. With such a workfunction difference, metals with higher electric Fermi level (*E*_F_) would donate electrons to the interfaced VO_2_ with lower *E*_F_ (Fig. 3c). Calculations show that one (1 × 1) VO_2_ unit could extract 0.47–2.50*e*^−^ from metals with lower workfunction (Fig. 3d; Supplementary Fig. 9; Supplementary Table 2). On the other hand, higher workfunction metals, Au and Pt, give nearly no extra electrons at the interface of M-VO_2_. It should also be noted in Fig. 3b that Al and Zn metals hold even lower workfunction than the lightly H-doped system of H_0.5_VO_2_, suggesting the continuing donation of electrons from metal to lightly hydrogenated VO_2_ which later attracts more hydrogen to penetrate. Therefore, the final products of Al/Zn-acid treatment are heavily H-doped VO_2_ with insulator phase while those of Ag/Cu-acid are conductive lightly H-doped VO_2_, as validated by XRD, XPS, XANES, and Raman characterizations in Supplementary Fig. 3. These results thus demonstrate that the electron-rich VO_2_ interface can attract and interact with the protons in acid solution, resulting in a feasible way to generate hydrogen atoms needed by hydrogenation.

**Fig. 4:**
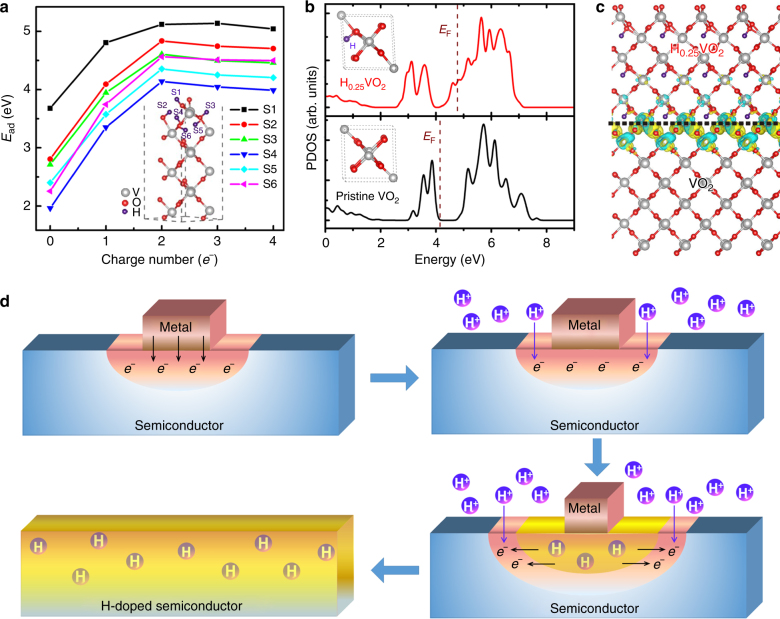
Electron–proton co-doping mechanism. **a** Computed adsorption energies for a proton to six adsorption sites of VO_2_ (020) surface, increased with the increasing amount of doped electrons. **b** Evolutions of V-3*d* partial density of state (PDOS), suggest the change of semiconductor band gap in the insulated pristine VO_2_ to the zero energy gap in H_0.25_VO_2_. Fermi level is marked with purple dashed lines. **c** Computed differential charge distribution at H_0.25_VO_2_–VO_2_ interface, showing each H_0.25_VO_2_ supercell donated ~2.06*e*^−^ to un-doped VO_2_. Here green and yellow bubbles represent hole and electron charges, respectively. **d** The schematic illustration of the contagious electron–proton co-doping mechanism with the metal-acid treatment to semiconductor: firstly the electrons flow to semiconductor when the metal contacts VO_2_ film. Once the metal/VO_2_ is immersing into acid solution, chemical reactions go sequentially as M – *x*[*e*^−^] → M^*x*+^ and VO_2_ + *x*[*e*^–^] + *x*[H^+^] → H_*x*_VO_2_. Here protons penetrate to meet electrons, creating conductive H-doped structure. Meanwhile the attached metal (M) is gradually dissolved in acid to become M^+^ cations for balancing charges in solution. Then the electrons flow from conductive H-doped structure to the un-doped parts, driving more proton penetration. Finally the repeated electron flowing–proton penetration–phase transition–electron further flowing cycle expands toward full H-doping

By examining six VO_2_ surface sites for a proton to adsorb (inset graph in Fig. 4a and Supplementary Fig. 10), it is found that more doped electrons lead to higher adsorption energies for all sites (Fig. 4a). For instance, on site 1, the proton adsorption energy of 3.68 eV in neutral circumstance is increased to 5.04 eV for a VO_2_ unit with 4*e*^−^ charge. The doped electrons also promote the diffusion of surface hydrogens into the VO_2_ crystal, with a possible migration pathway along the [100] direction (Supplementary Fig. 11). We can therefore propose an electron–proton co-doping strategy to create stable neutral H-doping in VO_2_. More specifically, driving by the electrostatic attraction, the surrounding protons could penetrate into VO_2_ to meet electrons, resulting in neutral H intercalation. The incorporation of H in the VO_2_ crystal prohibits further attack/adsorption of protons to oxygen, and increases the formation energy required for oxygen vacancy defect (Supplementary Fig. 12), resulting in the anti-corrosion ability in acid solutions.

The H-doping changes the VO_2_ electronic structures. For a VO_2_ unit with small H-doping concentration of H_0.25_VO_2_ (Fig. 4b), the evolution of the electronic structure is reflected by the computed partial density of state (PDOS) of the V-3*d* orbitals in Fig. 4b. The formation of H–O bonds causes electrons transferring from H to O atoms (Supplementary Table 3), which in turn promotes the electron occupancy of V-3*d* orbitals. Such effects give rise to the up-shifting of Fermi level from the pure VO_2_ to H_0.25_VO_2_ (Fig. 4b). Originally, VO_2_ exhibits a typical insulating state, with wide energy gap consisting of fully occupied valence band and empty conduction band. The H-doping then makes the conduction band edge states partially occupied, as for the case of H_0.25_VO_2_.

The same concept can be also used to explain the contagious hydrogenation process that enables a ~1 mm metal particle to convert a 2-inch semiconductor wafer. As shown in Fig. 3b, the work functions of the lightly hydrogenated H_0.25_VO_2_ with three facets of (020), (011), (100) are 4.32–4.96 eV, which are all lower than those of pristine VO_2_ around 5.12–5.85 eV. For any H-doped VO_2_ parts created by metal-acid treatment, electrons would flow/dope into neighboring unhoped VO_2_ with lower Fermi level (Fig. 4c; Supplementary Fig. 13). The electron-rich area drives further proton penetration to the neighboring un-doped VO_2_.

### Electron–proton co-doping mechanism

A contagious spreading of electron–proton co-doping mechanism is summarized in Fig. 4d: firstly the metal with lower workfunction donates electrons to the interfaced VO_2_ due to Fermi level difference, resulted in extra electrons accumulated in oxide layer; Then the doped electrons attract surrounding protons in acid solution to penetrate into the oxide semiconductor, creating H-doped structure at the top layer and causing the surface insulator-to-metal phase transition. Simultaneously, the attached metal particle is partially dissolved in acid, which balances the total charge in solution. This balance of charge is essential to drive the hydrogenation of VO_2_ as this route:

1 $${\mathrm{M}} - x[e^- ] \to {\mathrm{M}}^{x + },$$

2 $${\mathrm{VO}}_2 + x[e^ -] + x[{\mathrm{H}}^ +] \to {\mathrm{H}}_{x}{\mathrm{VO}}_2.$$

Otherwise, the reaction will follow the route of VO_2_ + 4H^+^ → V^4+^ + 2H_2_O, resulting the corrosion of VO_2_ in acid. The test of immersing only parts of a Cu/VO_2_ sample into acid without metal in solution causes no anticorrosive property (Supplementary Fig. 14), clearly showing this situation. After the above stage, the conductive H-doped structures delivery electrons to adjacent un-doped VO_2_ parts, triggering the next round of electron–proton co-doping and insulator-to-metal phase transition. Finally, the repeated electron flowing–proton penetration–phase transition–electron further flowing cycle expands toward full H-doped oxide material. This spreading of co-doping from metal center to edge is reflected by the onion-like contour map of resistance after metal-acid treatment (Fig. 2c), which decreases gradually from the metal-attached center to the wafer edge. In addition, the hydrogenation process is also gradually completed from the top to bottom layer with considering the time-dependent hydrogenation-related Raman or XRD signals (Supplementary Fig. 15).

It should be pointed out that since the corrosion of VO_2_ caused by oxygen atom moving out of lattice is much slower than the migrations of electrons or protons, the dynamics of this co-doping mechanism ensure the quick hydrogenation of VO_2_ surface before being corroded by acid, resulting in the anti-corrosion property of wafer-size VO_2_ film even at the beginning stage since we found the distinct resistance decrease of VO_2_ with several seconds metal-acid treatment.

## Discussion

On the basis of the proposed concept, one can anticipate that the metals with very low workfunction, such as Al or Zn, can lead to heavy hydrogenation of VO_2_ films in acid solution. It means that the induced metallic state would eventually be transferred into another new insulating state because of nearly saturated hydrogenation (Supplementary Fig. 3; Supplementary Fig. 16), which agree well with the different H concentrations revealed by secondary-ion mass spectrometry measurement (Supplementary Fig. 17). This observation is consistent with very recent findings of the consecutive insulator–metal–insulator transitions induced by increasing H-doping concentration^20^. In addition, based on this metal-acid treatment, partially hydrogenation process with a selected region can also be easily achieved by control the immersing area in acid solution (Supplementary Fig. 18). Remarkably, this simple metal-acid treatment is found to be a universal strategy that can be extended to doping ions in general. Replacing the acid solution by polymeric solution with Li^+^, metallic Li-doped VO_2_ films can also be obtained (Supplementary Fig. 19).

The ability to hydrogenate VO_2_ with protons in acid solution demonstrated here provides a facile strategy to induce phase modulation of VO_2_ materials, and the later successful doping of Li^+^ into VO_2_ suggests a general atomic doping approach of using proton or cation solvent sources together with electrons from metals. It is a sustainable approach that operates at ambient condition in an environment friendly manner by completely avoiding the use of precious catalysts and high-energy consumptions. The doping concept established in this study will have strong impact on the development of new functional materials in different applications.

## Methods

### Thin-film growth

The 2-inch wafer-size VO_2_ (020) epitaxial films were grown on *c*-cut sapphire by an rf-plasma assisted oxide molecular beam epitaxy (rf-OMBE) equipment and more details for the film preparation are reported elsewhere^29^.

### Conventional hydrogenation treatment for VO_2_ film

Nano-sized Au islands were sputtered on M-VO_2_ surface as the catalysis. The Au/VO_2_ samples were annealed in tube furnace with the forming gas (15% H_2_/85% Ar) under various conditions. The lightly doped metallic H-VO_2_ (120 °C for 2 h) and heavily doped insulated H-VO_2_ (180 °C for 10 h) were prepared.

### Characterizations

The resistance as the function of temperature was examined by Keithley 2400 sourcemeter with a variable temperature stage. For all of the measurements, the temperature sweeping rate was set at 0.1 K/s; The resistance distribution mapping for the prepared wafer-size VO_2_ film were tested on room temperature and 120 °C, respectively. The cross-section and surface morphologies were investigated by Scanning Electron Microscopy (Gemini Fe-SEM 500 and FEI Sirion 200). Raman spectra were recorded by an integrated laser Raman system (LABRAM HR, Jobin Yvon). The 632.8 nm He–Ne laser was used as the excitation source. To obtain direct information about the hydrogen concentration of the metal-acid VO_2_ film sample, the secondary-ion mass spectrometry (SIMS) measurements (Quad PHI6600) were conducted. The ICP-AES equipment (Optima 7300DV) was used to trace element concentration. The wavelength of Cu, V adopted 327.393, 290.880 nm respectively. The emission power is 1250 w. The distinguishability reached to 0.003 nm at 200 nm, and the detection limit is 4 ng/ml.

### Synchrotron-based measurements

Synchrotron X-ray diffraction spectra, including *θ − *2*θ*, X-ray reflectivity (XRR), *Φ*-scan, rocking-curve, were conducted at the BL14B beamline of the Shanghai Synchrotron Radiation Facility (SSRF). The SSRF is a third-generate accelerator with a 3.5 GeV storage ring. The BL14B beamline shows the energy resolution (Δ*E*/*E*) of 1.5 × 10^−4^ @10 keV and the beam size of 0.3 × 0.35 mm with the photo flux of up to 2 × 10^12^ phs/s@10 keV. Considering the photo flux distribution and the resolution, the 0.12398 nm X-ray was chosen during the experiment.

The X-ray absorption near-edge spectroscopy (XANES) was conducted at the XMCD beamline (BL12B) in National synchrotron radiation laboratory (NSRL), Hefei. The total electron yield (TEY) mode was applied to collect the sample drain current under a vacuum better than 3.75 × 10^−10^ Torr. The energy range is 100–1000 eV with an energy resolution of ca. 0.2 eV. The X-ray incident angle is 54.7°. During the measurement, the samples were firmly adhered on the conductive substrate with random orientation, so the polarization dependence is not considered.

The X-ray photoelectron spectroscopy (XPS) beamline was conducted in National synchrotron radiation laboratory (NSRL), Hefei. The photoemission beamline covers photon energies from 100 to 1000 eV with a typical photon flux of 1 × 10^10^ phs/s and a resolution (*E*/Δ*E*) better than 1000 at 244 eV. The analysis chamber is connected to the beamline and equipped with a VG Scienta R3000 electron energy analyzer, and the base pressure is 1.5 × 10^−10^ Torr.

### First-principles calculations

All calculations are performed with density functional theory (DFT), using the Vienna ab initio simulation package (VASP) code^33^. The exchange and correlation terms are described using general gradient approximation (GGA) in the scheme of Perdew–Burke–Ernzerhof (PBE)^34^. Core electrons are described by pseudopotentials generated from the projector augmented-wave method^35^, and valence electrons are expanded in a plane-wave basis set with an energy cutoff of 480 eV. Slab model method is used to model the VO_2_ surface and metal–VO_2_ interface, the thickness of vacuum are larger than 15 Å. The DFT+*U* method is employed to optimize the structure, *U* and *J* are chosen to be 4 and 0.68 eV. The geometry relaxation is carried out until all forces on the free ions are converged to 0.01 eV/Å. In the calculation of electronic structures, DFT with hybrid functionals proposed by Heyd, Scuseria, and Ernzerhof (HSE06) is used^36^. Climbing image nudged elastic band (CI-NEB) method^37^ is used to find the minimum energy paths and the transition states for diffusion of H from surface to subsurface, with a force converge <0.05 eV/Å.

### Data availability

The remaining data contained within the paper and supplementary files are available from the authors upon request.

## Electronic supplementary material

Supplementary Information

Peer Review File
