# Supplementary material for: Non-catalytic hydrogenation of VO2 in acid solution
Source: Nat Commun. 2018 Feb 26;9:818. doi: 10.1038/s41467-018-03292-y (PMC5827755; doi:10.1038/s41467-018-03292-y)
Supplement: Supplementary file 1 — Supplementary Information [file 41467_2018_3292_MOESM1_ESM.pdf]

Supplementary Information for "Non-catalytic Hydrogenation of VO_2 in Acid Solution"

Chen *et al.*

Supplementary Figures

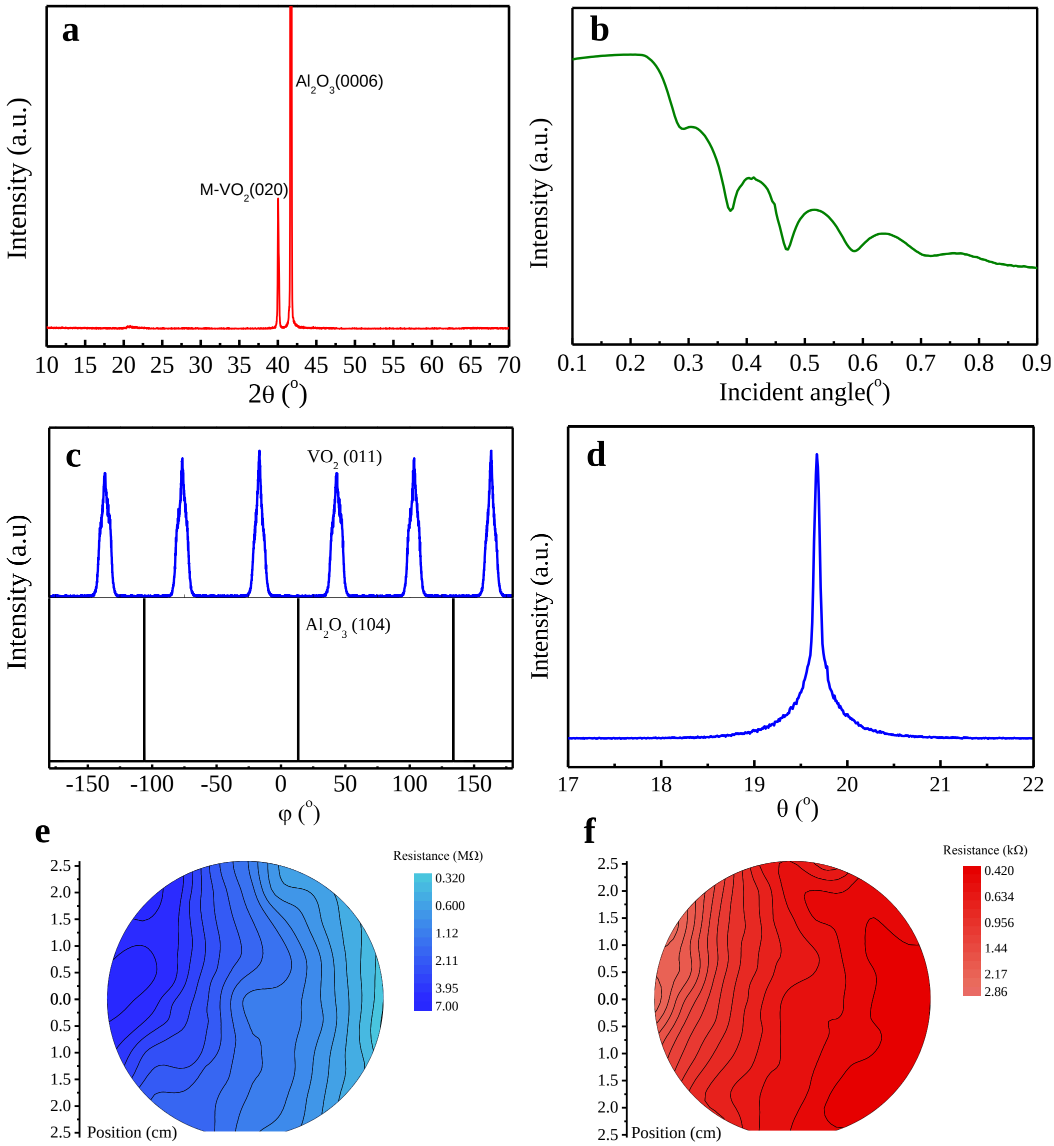

Supplementary Figure 1 | VO_2 epitaxial film Characterizations. a, The *θ-2θ* scan XRD for the 2-inch M-VO_2/Al_2O_3 film, the unique diffraction peak of (020) shows the preferred growth orientation. **b,** The XRR for the M-VO_2 film with pronounced oscillation peaks, showing the smooth and flat film surface. **c**, The *Φ*-scan mode diffraction shows the epitaxial growth of VO_2 layer on Al_2O_3(0001) surface. **d**, The rocking-curve of the M-VO_2/Al_2O_3 film; **e** and **f**, The surface resistance distributions of the 2-inch M-VO_2/Al_2O_3 film during the temperature triggered MIT phase transition. It is clear that above the critical temperature of ~68°C, the resistance still is ladder-distribution consistent with primary resistance.

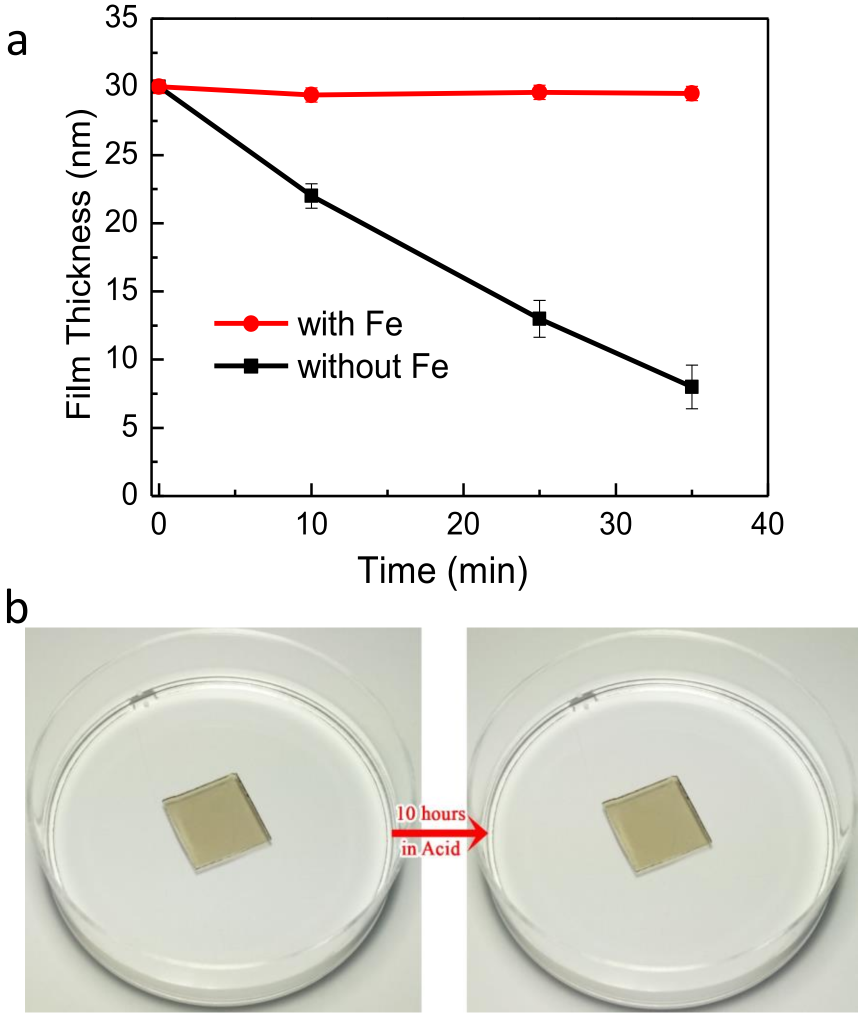

Supplementary Figure 2 | Excellent anti-corrosion performance hydrogenated VO_2 film. a, VO_2 film thickness measured by AFM as the function of immersing time in acid solution with and without metal contact. It can be observed that under the metal-acid condition, the film thickness has almost no change. While without the metal (Fe) contact, the film thickness is gradually decreasing due to the corrosion of VO_2 in acid solution. **b**, The M-VO_2/Al_2O_3 film hydrogenated by Au catalysis at 120°C, remained intact after being immersed into 2%wt H_2SO_4 acid solution for 10 hours. In addition, we have conducted the metal-acid treatment in 10%wt and up to 20%wt H_2SO_4 acid, which shows that the anti-corrosion property is still observed. We have also tested the experiments by dilute hydrochloric acid or oxalic acid, which show the similar results.

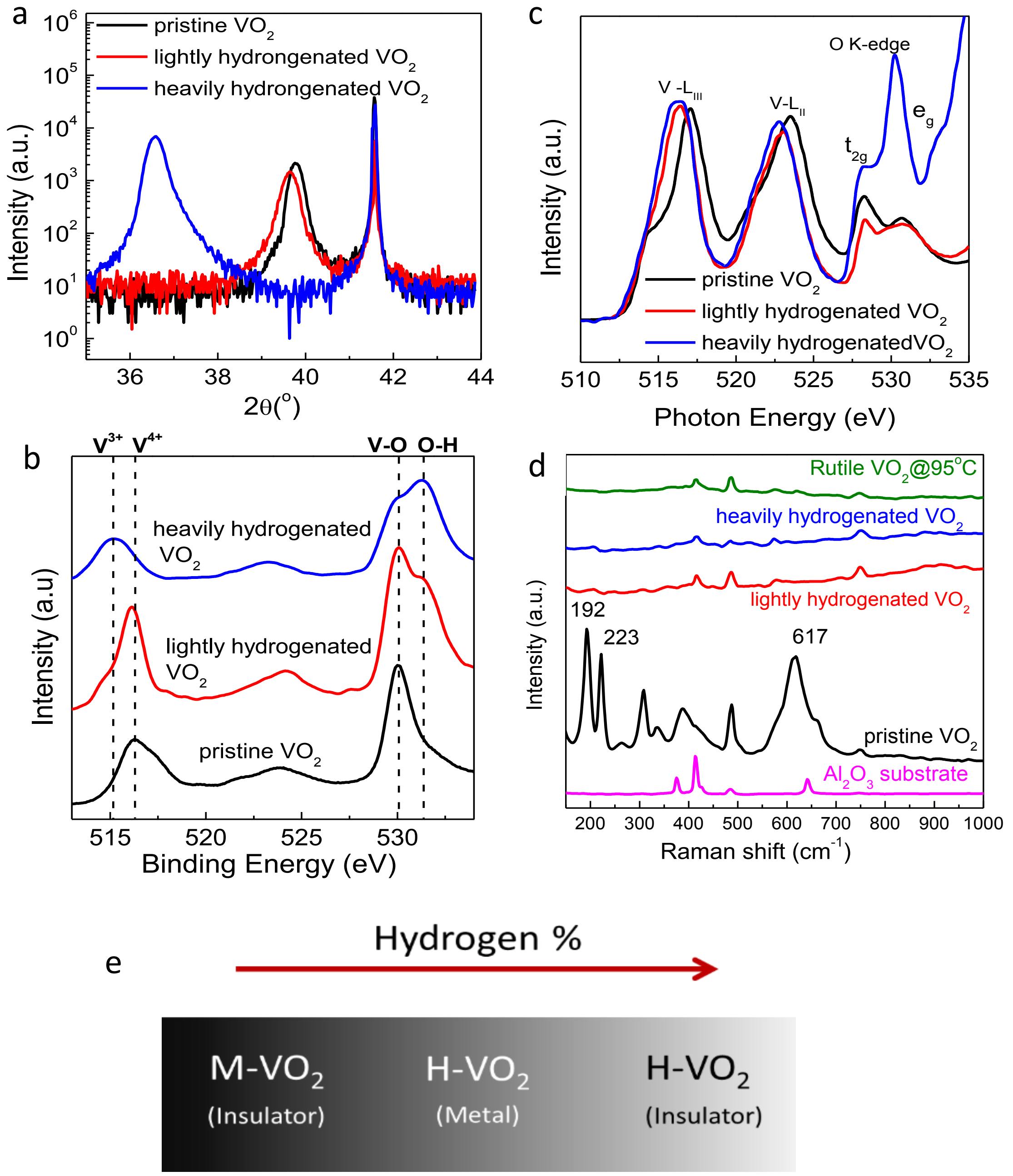

Supplementary Figure 3 | Structural and electronic properties for hydrogenated VO_2 film by Au catalysis at high temperature. a, XRD tests, **b**, XPS tests, **c**, XANES tests and **d**, Raman spectra characterizations for the pristine VO_2 sample as well as the lightly (metallic) and heavily (insulator) hydrogenated samples. In XRD curves, the pronounced (020) XRD peak shift from 39.8° to 36.7°, the increased V^{3+} and O-H XPS signals, and the variation of electron occupancy as reflected by increased e_g/t_{2g} XANES signal ratio along with increasing metal-acid time, all indicated lattice changes and O-

H bonds formations due to light and heavy hydrogenations. The Raman measurement showed that the Raman peaks at 192, 223 and 617 cm^{-1} in pristine M-VO_2 were completely disappeared, similar as the test from Rutile VO_2 phase. From the above tests, it is suggested that the hydrogenated VO_2 film shows a rutile-like phase structure. **e**, the scheme of hydrogenated VO_2 film, which shows successive metallic state and insulator state as the hydrogen doping concentration increasing.

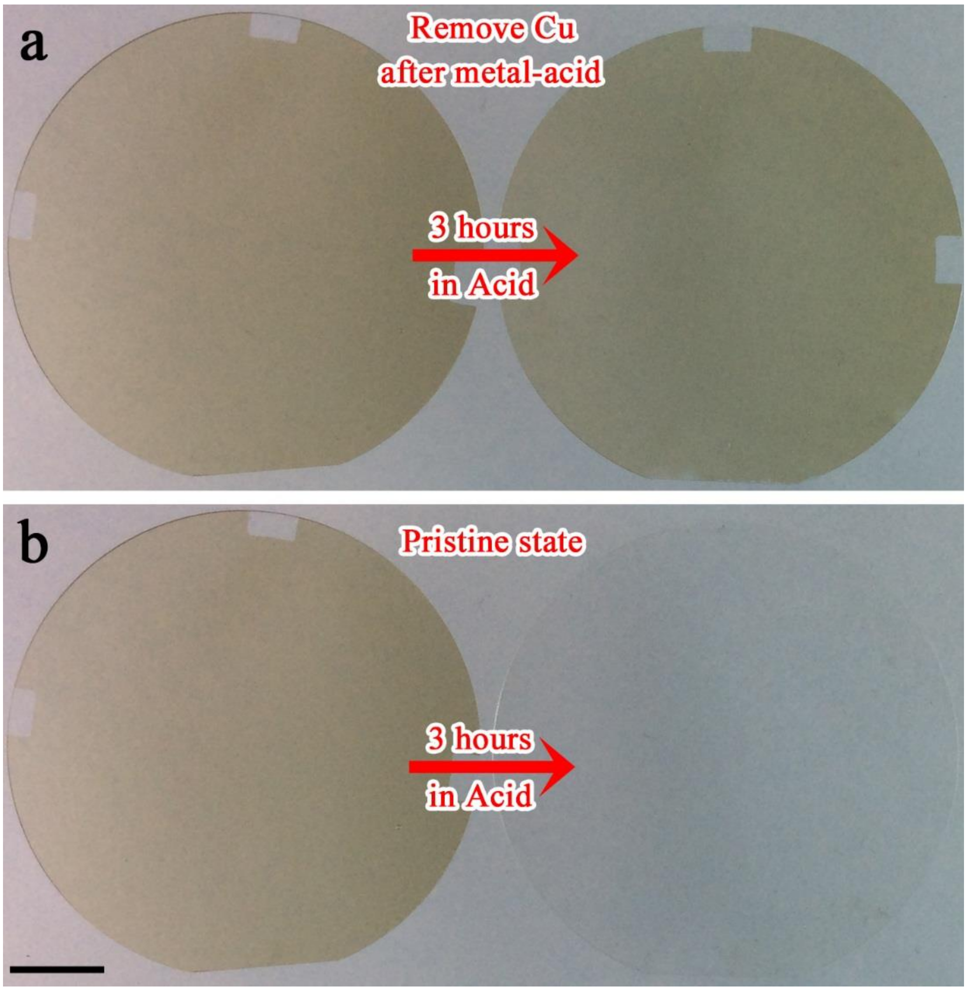

Supplementary Figure 4 | Anti-corrosion performance of the acid-metal treated VO_2 film. a, The hydrogenated VO_2 film (H-VO_2) induced by the metal-acid treatment demonstrated strong anti-corrosion ability even after taking away the Cu attachment. **b**, The pristine M-VO_2 film is corroded in H_2SO_4 solution completely after 3 hours. The scale bar is 1.0cm.

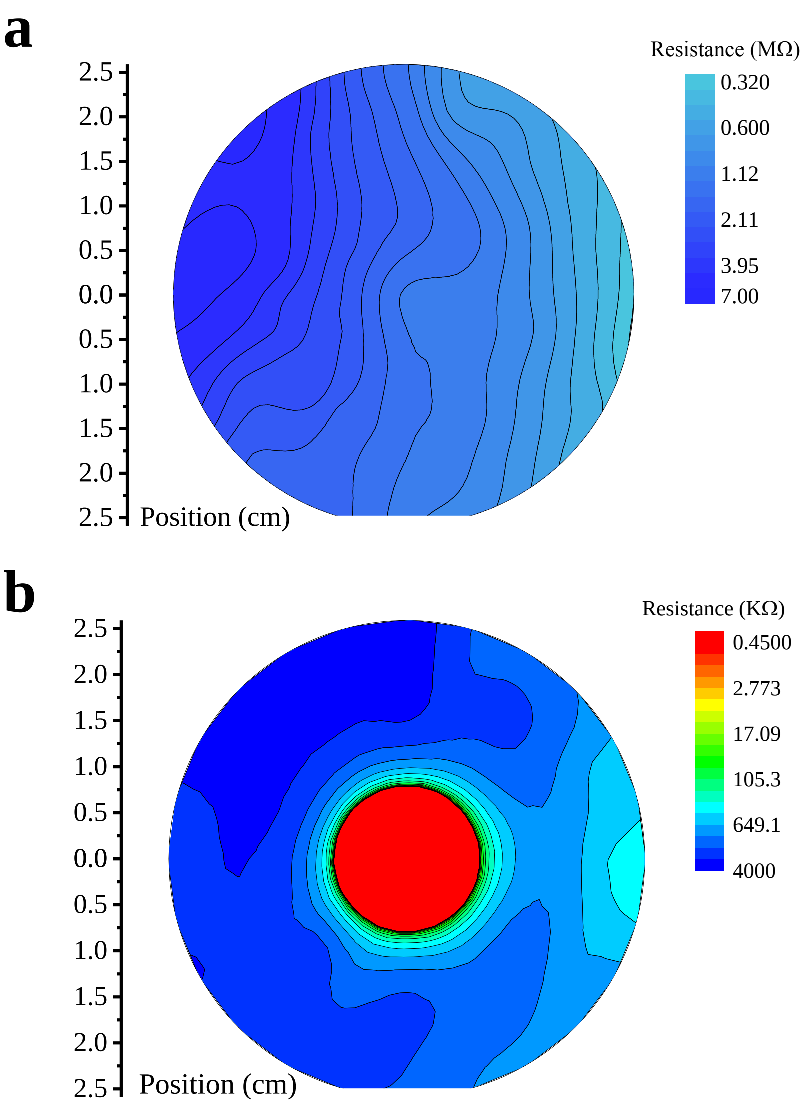

Supplementary Figure 5 | Resistance change of hydrogenated VO_2 film through conventional noble-metal catalysis. a and **b**, The surface resistance distributions of 2-inch M-VO_2/Al_2O_3 film before and after the conventional hydrogenation treatment assisted by Au as the catalysis at 120°C. The Au particles was deposited on the center round area within a 1.5cm diameter. It is observed that the hydrogenation is only available in the Au neighboring area due to the limited diffusion distance of H atoms.

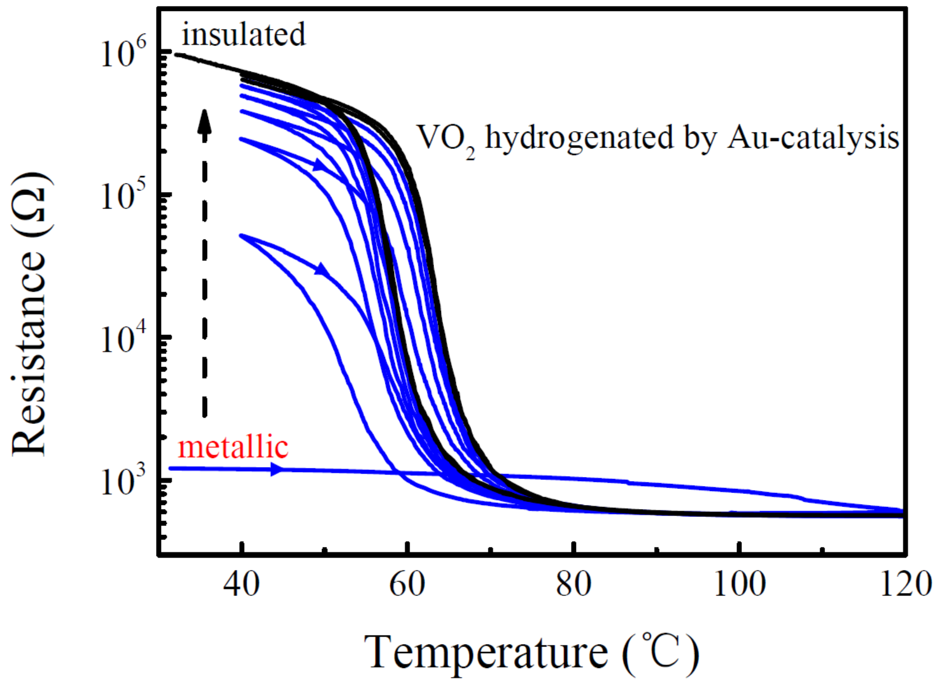

Supplementary Figure 6 | Recovery of monoclinic VO_2 from metallic hydrogenated VO_2. The surface resistance of the metallic hydrogenated H-VO_2/Al_2O_3 film (through conventional Au catalysis at 120°C) were increased to the level of insulated pristine VO_2 after being heated.

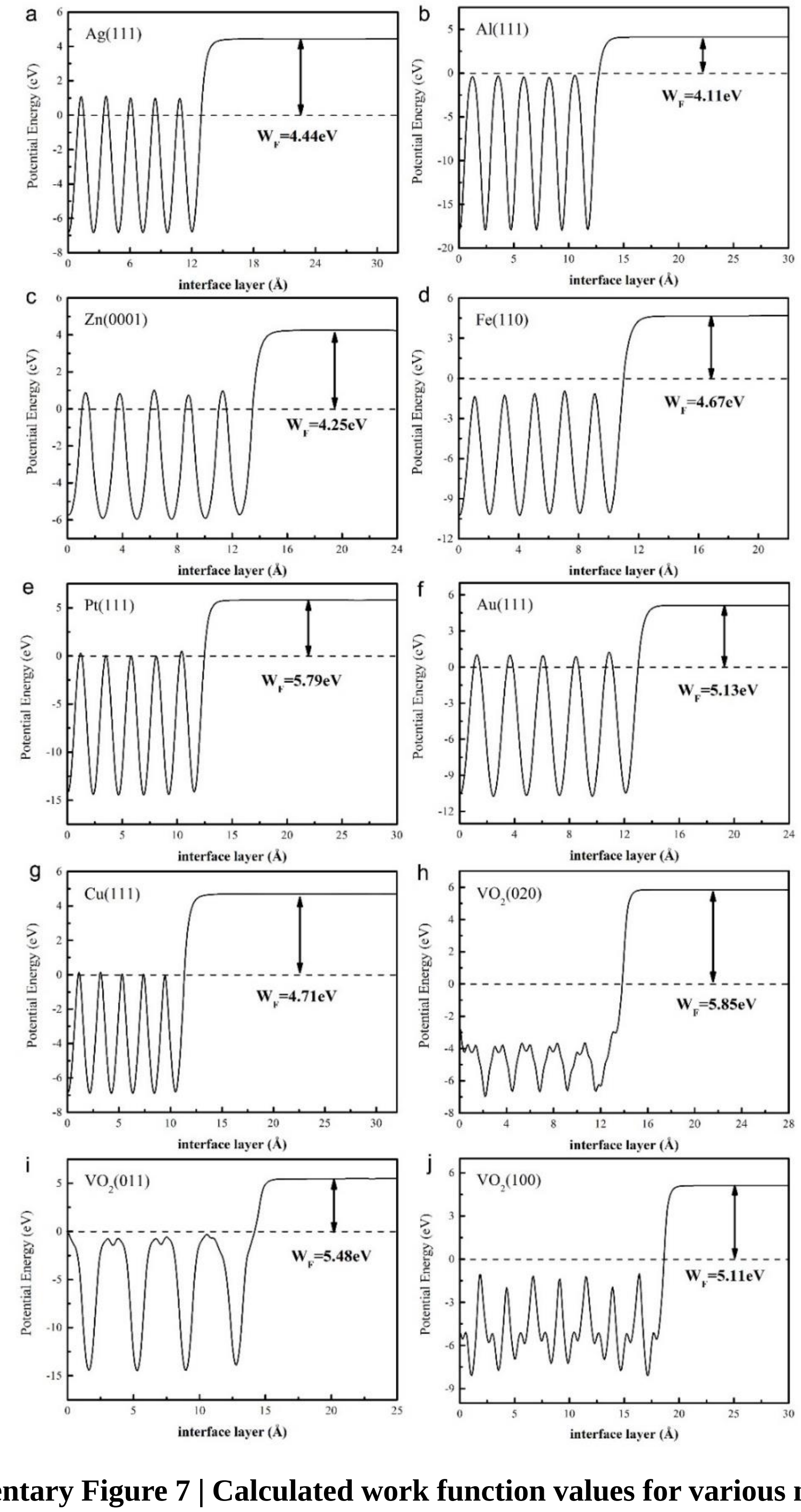

Supplementary Figure 7 | Calculated work function values for various metals and VO_2 crystal. Computed potential surfaces with work function (W_F) values for **a,** Ag(111); **b,** Al(111); **c,** Zn(0001); **d,** Fe(110); **e,** Pt(111); **f,** Au(111); **g**, Cu(111); **h,** VO_2(020); **i,** VO_2 (011) and **j,** VO_2 (010). The Fermi levels are set to zero.

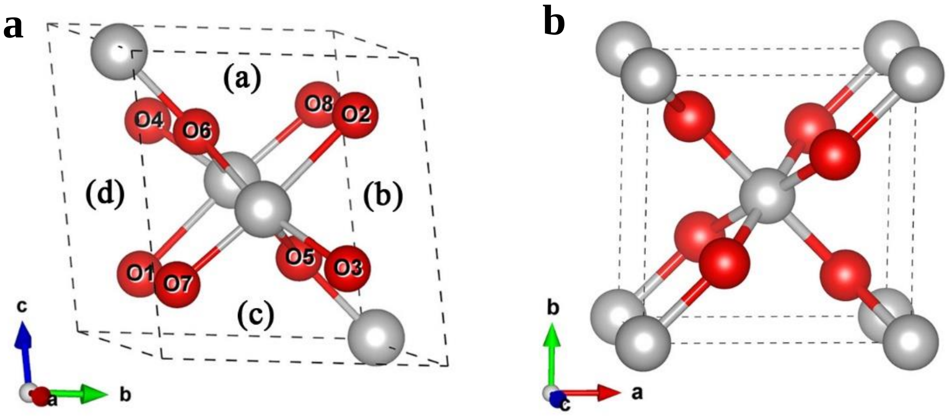

Supplementary Figure 8 | VO_2 crystal lattices. **a**, The atomic models of the monoclinic VO_2 unit cell. For monoclinic phase, the O atoms in the unit are not equal. Here we divide the unit into four regions and the H atom bonding to atom Ox (x=1-8) in the corresponding interstitial site is marked as H(a), H(b), H(c) and H(d). **b**, The atomic models of rutile VO_2 unit cell. For rutile phase, all O atoms are equal because of high symmetry, so there is actually only one interstitial site for H in a V_2O_4 unit cell.

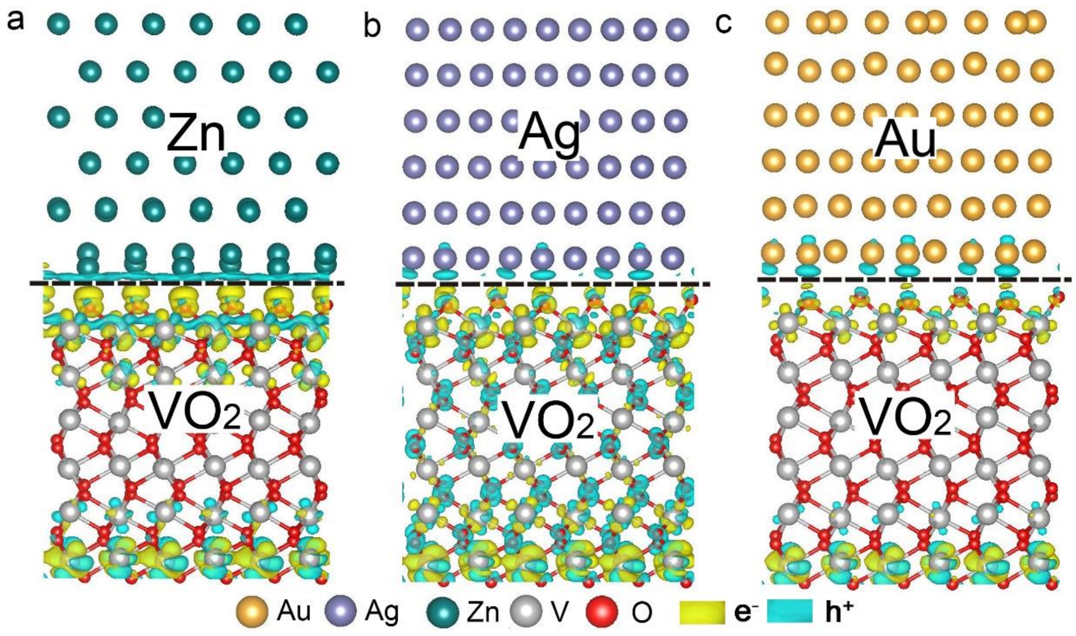

Supplementary Figure 9 | Charge distributions between different metal and VO_2 (020) surface. Computed differential charge distributions at Zn/Ag/Au-VO_2(020) interface, indicating free electrons migrate from Zn/Ag into VO_2, while Au cannot.

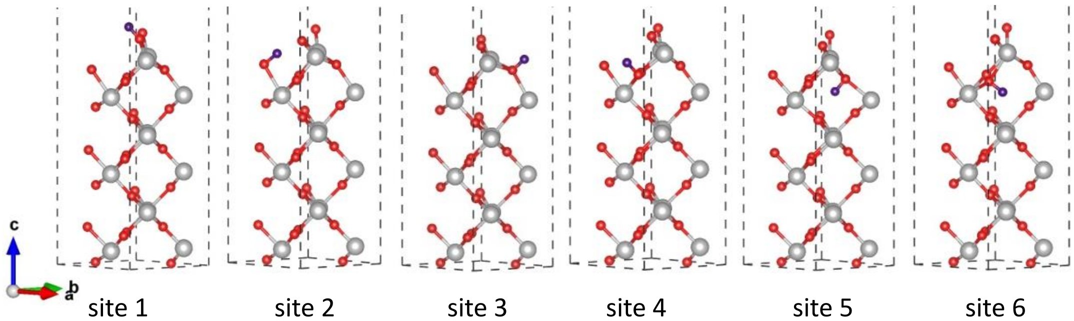

Supplementary Figure 10 | Six hydrogen adsorption sites on the VO_2 (020) surface. These structures were optimized in neutral circumstance. The adsorption energy of H^+ on the charged VO_2 (020) surface is calculated as:

$$E_{adsorption} = E_{(VO_2)^{-xe}} + E_{H^+} - E_{(H-VO_2)^{(1-x)e}} \qquad (1)$$

Where $E_{adsorption}$ is the adsorption energy of H^+ ion. $E_{(VO_2)^{-xe}}$ and $E_{(H-VO_2)^{(1-x)e}}$ stand for the energies of charged VO_2 surface and that of charged adsorption system. E_{H^+} is the energy of a H^+ ion which is set to be zero.

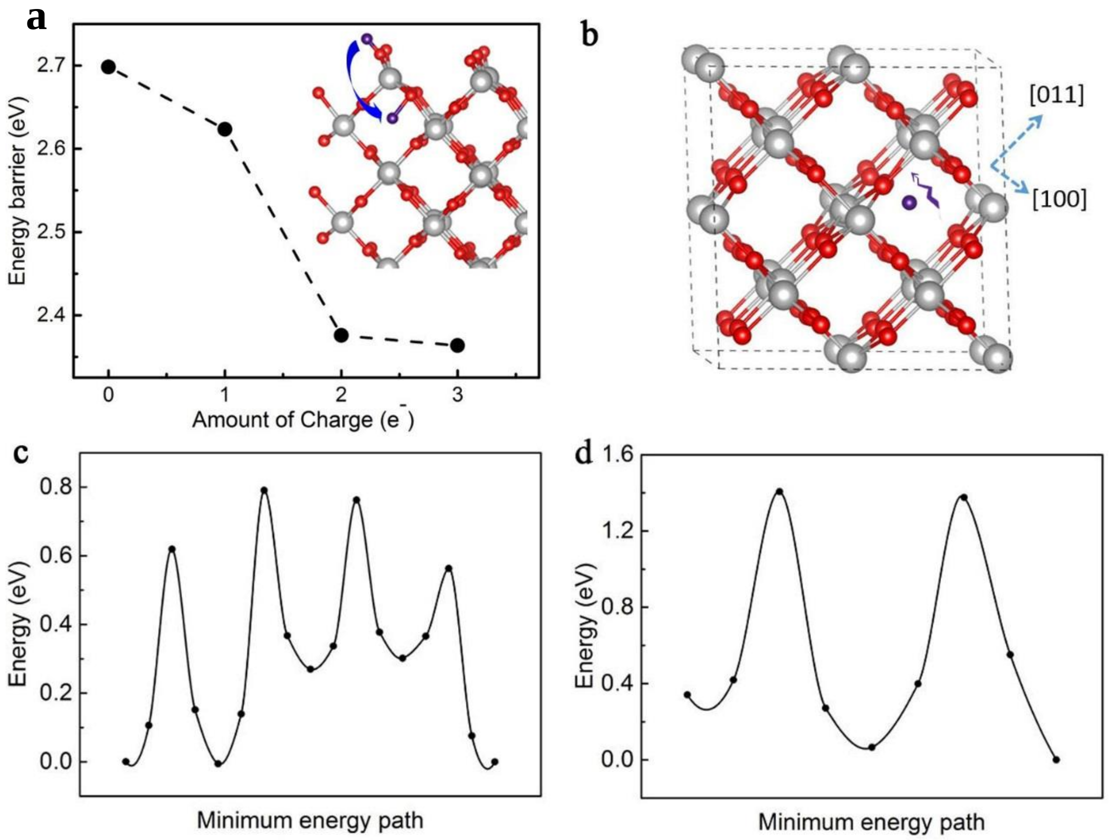

Supplementary Figure 11 | Dependence of proton diffusion energy barrier and migration pathway in VO_2. a, The diffusion pathway from surface to subsurface is depicted in the inset graph. Gray, red, purple beads stand for V, O, H atoms, respectively. Focusing on the first diffusion step from surface to subsurface (inset graph), the diffusion energy barrier decreased with the increasing of doped electrons in VO_2. **b,** The H migration pathways along [100] direction as found by NEB transition state simulations. **c**, The energy profile of the diffusion pathway along the [100] direction. **d**, The energy profile of the diffusion pathway along the [011] direction. Using the nudged elastic band (CI-NEB) calculations for transition states, we have examined in theory the H migration pathway in VO_2. From the energy profiles of H diffusion along the [100] and [011] direction, we found the energy barrier values as 0.8 and 1.4 eV, respectively. This suggests a most likely H migration pathway along the [100] direction.

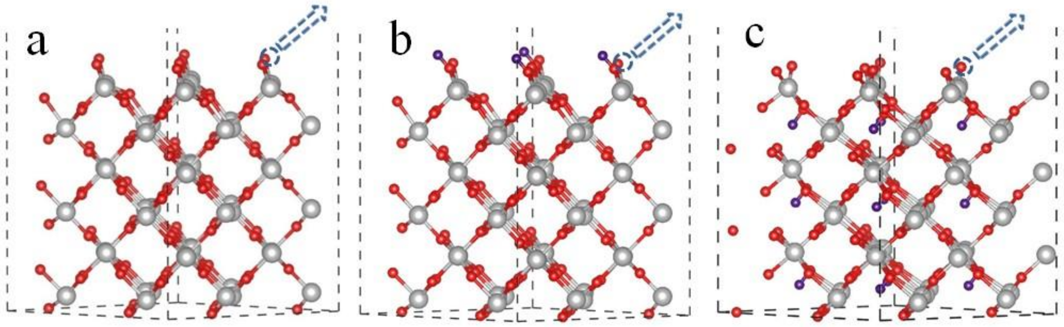

Supplementary Figure 12 | Schematic diagram for the formation of an oxygen vacancy defect on different VO_2 surface. **a,** VO_2 surface. **b**, H-absorbed VO_2 surface and **c**, H-doped VO_2 ($H_{0.25}VO_2$) surface. From microscopic view, the corrosion of VO_2 starts from the attack of protons to oxygen, which breaks O-V bonds and creates O vacancy (OV) defect on surface. Obviously, the presence of H on surface or inside bulk VO_2 will change the formation energy (E_f) of OV on the surface. OVs are created in three different situations as shown in (**a**, **b** and **c**). The formation energy E_{OV} of an oxygen vacancy can be expressed as

$$E_{OV} = E_{vac} - E_{st} + \frac{1}{2} E_{O_2} \qquad (2)$$

where E_{st} and E_{vac} stand for the energy of the stoichiometric structure and that of the same structure except containing an OV, respectively, and E_{O2} is the energy of an isolated O_2 molecule. The E_{OV} of OV on clean VO_2 (020) is 3.88 eV (**a**). When H atoms are adsorbed on the surface, the E_{OV} is increased to 4.93eV (**b**). The E_{OV} of H-doped VO_2 (HV_4O_8) surface (**c**) are 4.05eV. These results suggested that both the adsorption of H on the surface and incorporation of H in the bulk increase the formation energy of OV and thereby protect VO_2 from corrosion in acid solutions.

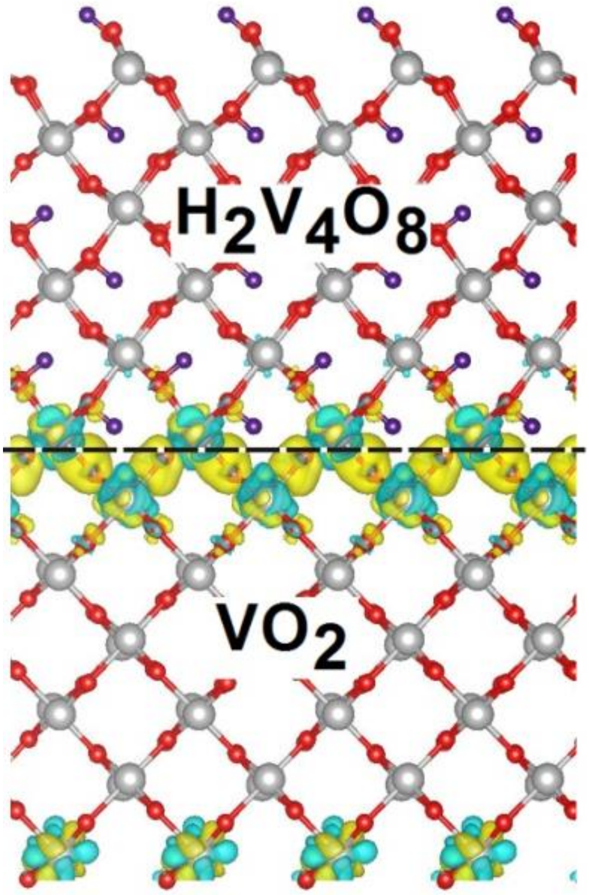

Supplementary Figure 13 | Computed differential charge distribution at $H_{0.5}VO_2$-VO_2. It shows that the $H_{0.5}VO_2$ in each supercell donates about 2.12 electrons to VO_2.

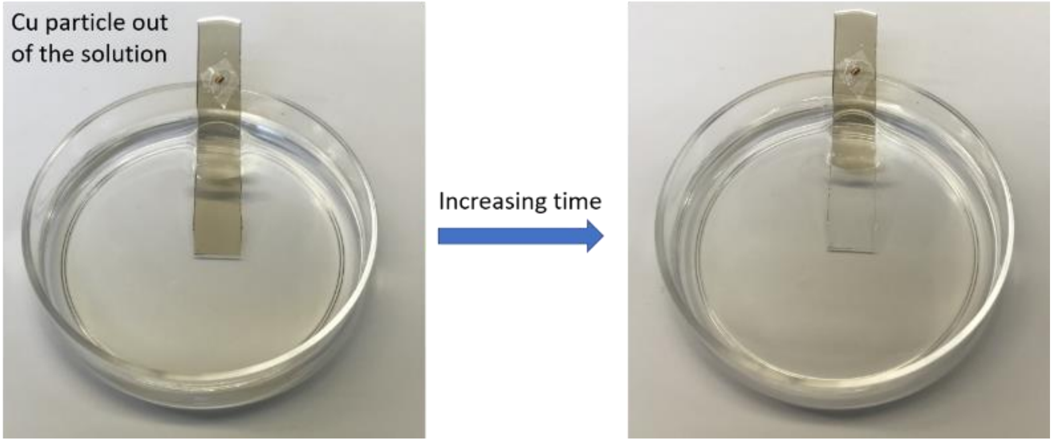

Supplementary Figure 14 | No anti-corrosion property if immersing only parts of a Cu/VO_2 system into acid without metal in solution. This test shows that if we put half of the film contacted with Cu metal left outside of the liquid, the other half from the same piece of film in the acid will be dissolve (corroded) soon. It indicates that the balancing of charges in solution is essential for hydrogenating VO_2 in the metal-acid treatment through:

$M\text{-}x[e^-] \rightarrow M^{x+}$ (3)

$VO_2 + x[e^-] + x[H^+] \rightarrow H_xVO_2$ (4)

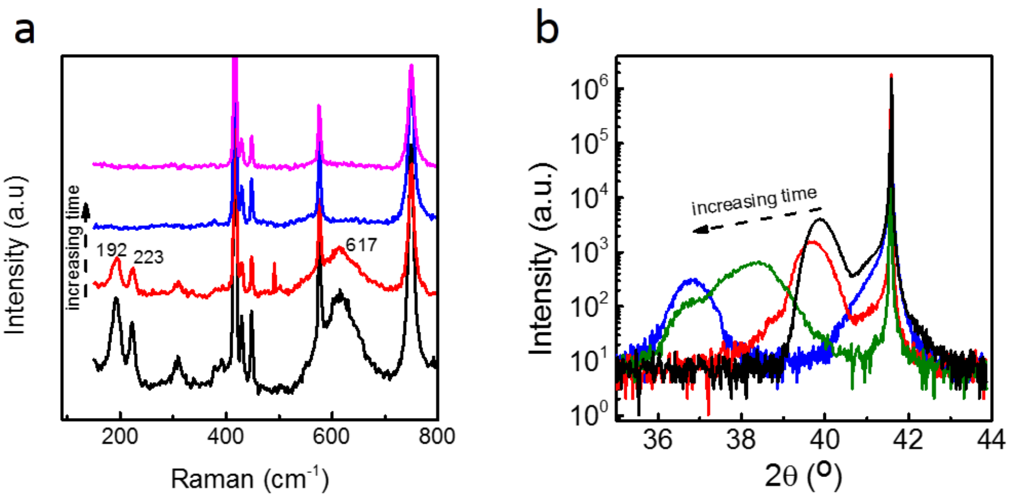

Supplementary Figure 15 | Time-dependent characterizations. a and b, Raman and XRD results for the samples tests with different meta-acid treatment time**.** It is observed that as the meta-acid treatment time increasing, the M-VO_2 film was actually converted gradually to the fully hydrogenated state. From the variation of Raman peak intensity as well as the shift of XRD, it is deduced that the hydrogenation process is gradually completed from top to bottom parts.

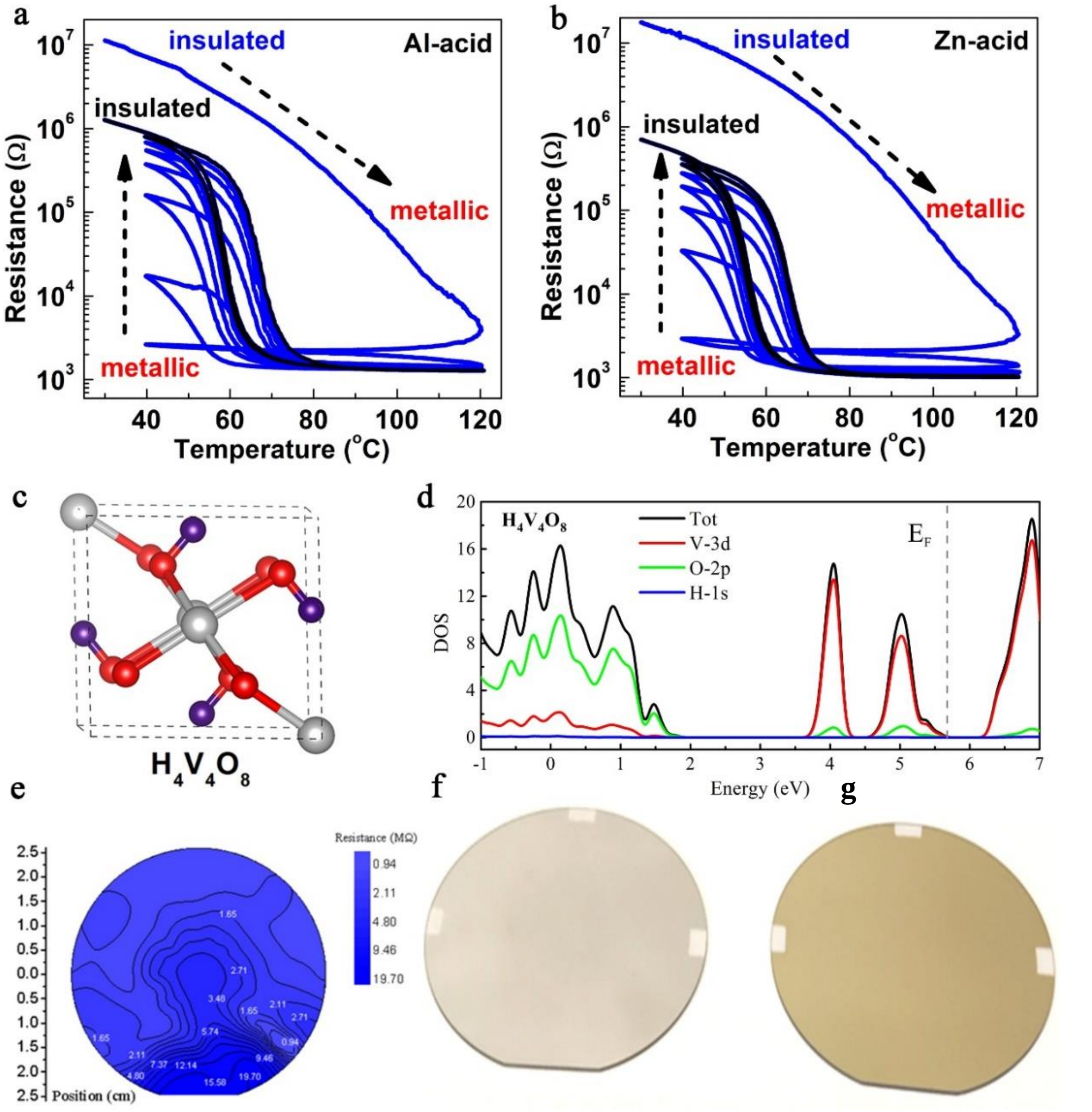

Supplementary Figure 16 | Saturated hydrogenation creates insulated H-VO_2. a and **b**, The VO_2 sample hydrogenated by metal-acid treatment with Al and Zn , exhibited another new insulating phase, when H-incorporation are saturated in the VO_2 lattice. In the experiment, we hydrogenated two VO_2 film samples by metal-acid treatment with Al and Zn at room temperature respectively. After quite long immersing time, the two samples were found to be fully hydrogenated into the insulating phases. Then we take them out of acid solution, and conduct the Resistance-Temperature measurement by ramping the temperature. It can be observed that this H-doped insulating state will be gradually converted to metallic H-doped phase and finally go back to the original insulating M-VO_2 due to the hydrogen desorption from VO_2 crystal during the R-T testing cycles. **c**, The crystal lattice of the heavily hydrogenated system

of $H_4V_4O_8$; **d**, The energy band gap of the heavily hydrogenated system of $H_4V_4O_8$, showing the insulated property. **e**, The resistance mapping of the fully hydrogenated insulating wafer; **f** and **g**, the optical pictures for the fully hydrogenated insulating wafers and pristine M-VO_2 wafer respectively.

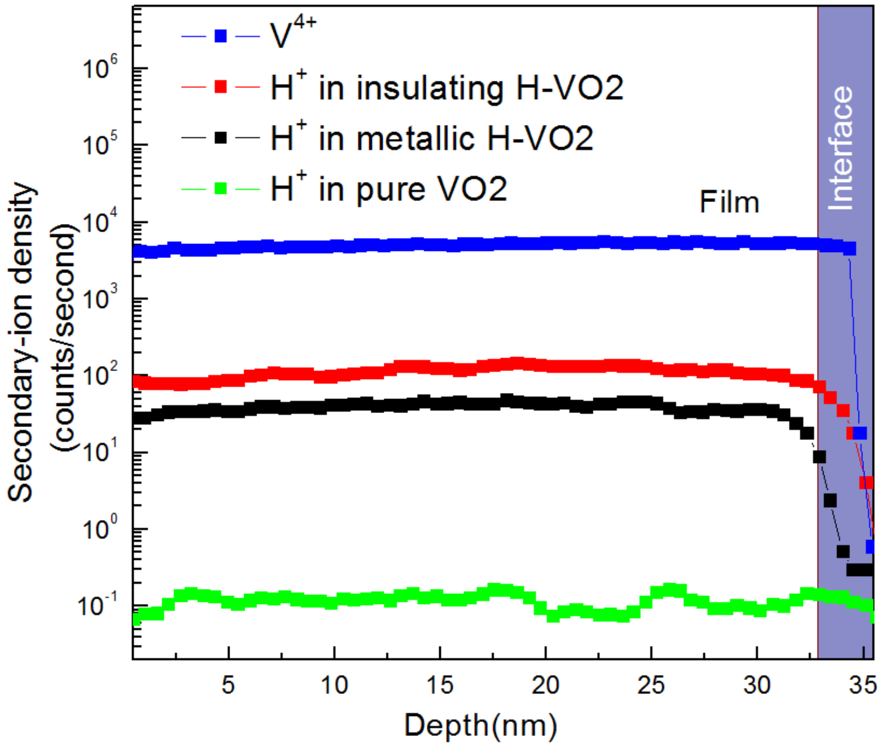

Supplementary Figure 17 | SIMS test for hydrogenated VO_2 films. The depth profiles of H^+ and V^{4+} ions from the pure VO_2 film and the hydrogenated VO_2 films were recorded by secondary-ion mass spectrometry (SIMS). It was observed that quite low H atoms concentration was tested for the pure VO_2 film. While for the metal-acid treated VO_2 films, the H atoms concentration was much higher. It was clear that the insulator H-VO_2 showed higher H atoms doping. The interface between the VO_2 film and substrate can be clearly observed according to the H^+ and V^{4+} ions curves.

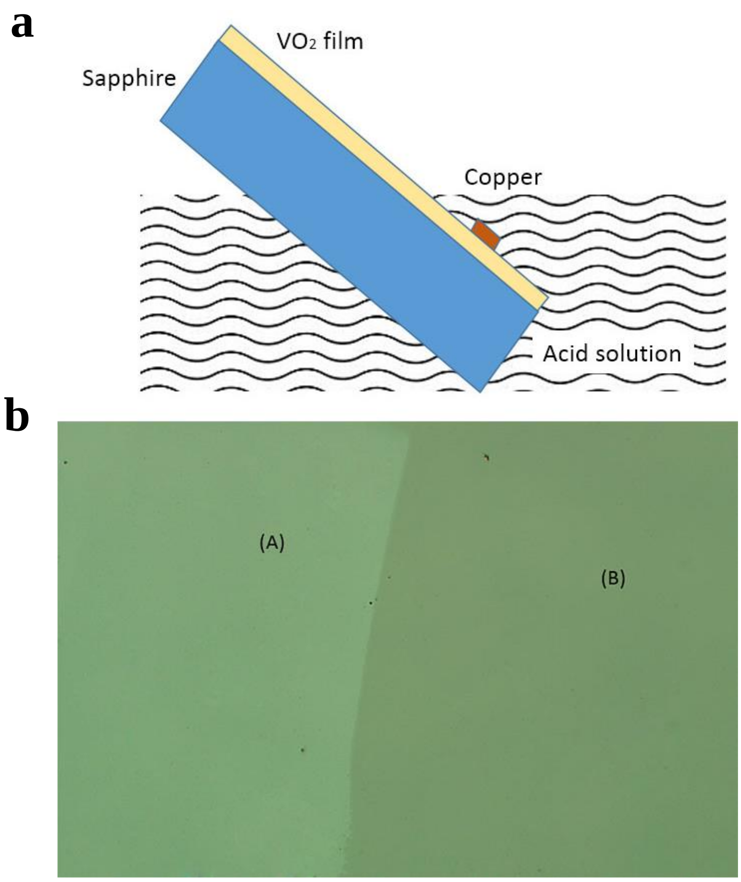

Supplementary Figure 18 | Selected hydrogenation by metal-acid treatment. a, The scheme for the partially hydrogenation process by the way of acid treatment; **b**, The optical microscopy image of the M-phase VO_2 film (A) and the H-VO_2 (B). The boundary can be clearly observed.

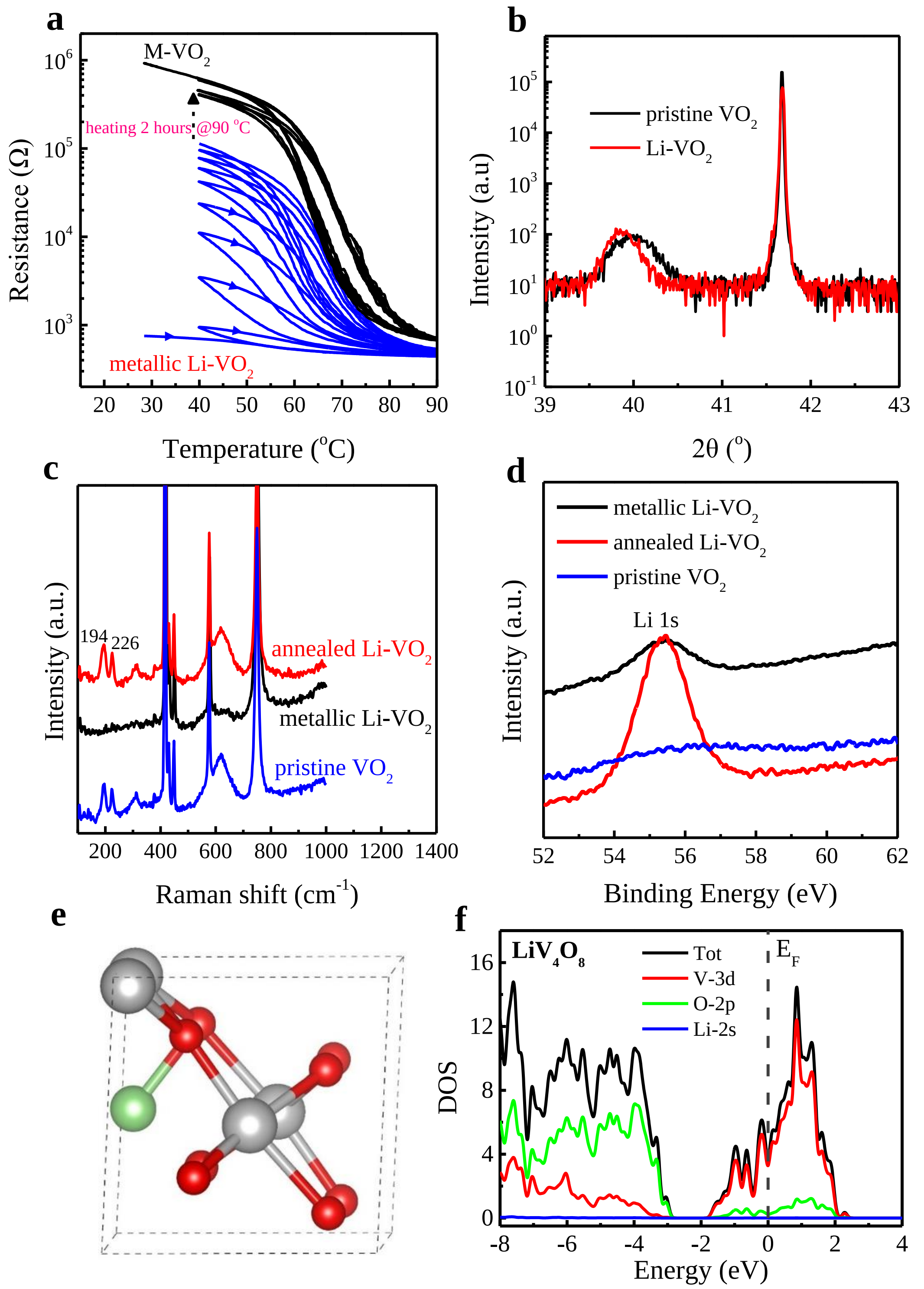

Supplementary Figure 19 | Li-doped VO_2 film through the metal-Li^+ solution treatment. This experiment was conducted in a vacuum condition (<7.5 × 10^{-3}Torr), avoiding the effect of moisture in air. Specifically, a prepared VO_2 film attached with an Al particle was immersed in saturated lithium perchlorate/propylene carbonate solution. **a,** The R-T test for the metallic Li-VO_2 film. It can be observed that after cycles of R-T tests, the films will gradually recover to the initial M-VO_2 with distinct

MIT property. After heating the sample in air for 2 hours at 90°C, the film completely restored to M-VO_2. **b,** XRD curves for the M-VO_2 and Li-VO_2 samples. The VO_2 (020) diffraction peak shift towards low angle direction, indicating the expanding of the unit cell due to the Li intercalation. **c,** Raman spectra for the M-VO_2 and metallic Li-doped VO_2 sample. **d,** XPS for the metallic Li-VO_2 sample and the annealed Li-VO_2 sample. A small Li-1s peak exists for Li-VO_2 film, while this peak becomes much stronger after annealed, showing the Li atoms are driven out from the VO_2 lattice and congregated on the surface to form some Li-oxides. **e,** The atomic structure of Li-doped VO_2 crystal. **f,** The calculated DOS for the Li-doped VO_2, showing the metallic state.

Supplementary Tables

Supplementary Table 1. The computed total energy (eV) of a monoclinic V_4O_8 unit cell with H-doping at 16 different sites and a rutile unit with one H-doping site (see Supplementary Figure 8), from which we extracted the one with the lowest energy (O4-H(d)) for further electronic structure and property simulations of monoclinic V_4O_8 unit.

System	O1-H(c)	O1-H(d)	O2-H(b)	O2-H(a)
Energy(eV)	-106.21981	-106.15082	-106.15079	-106.21986
System	O3-H(c)	O3-H(b)	O4-H(d)	O4-H(a)
Energy(eV)	-106.15082	-106.21984	-106.21988	-106.15073
System	O5-H(c)	O5-H(b)	O6-H(d)	O6-H(a)
Energy(eV)	-106.16119	-106.21987	-106.21985	-106.16127
System	O7-H(c)	O7-H(d)	O8-H(a)	O8-H(b)
Energy(eV)	-106.21987	-106.16126	-106.21981	-106.16123
System	O-H Rutile			
Energy(eV)	-54.679315			

Supplementary Table 2 | The amount of electron flowing from metal into a (1×1) unit of VO_2 when metals are contacted to VO_2 (020) or VO_2 (100).

interface	Cu/VO_2 (020)	Ag/VO_2 (020)	Al/VO_2 (020)	Zn/VO_2 (020)	Au/VO_2 (020)	Pt/VO_2 (020)	Pt/VO_2 (100)
Extra Charge to VO_2 (e^-)	0.78	0.47	2.50	0.64	0.07	0.13	-0.06

Supplementary Table 3. The computed H-doping induced charges on each atom (subtract the Bader charge of pure V_4O_8 unit cell from the doped unit) in three H-doping monoclinic VO_2 cells. For H atom, the value is subtracted from an isolated hydrogen.

	HV_4O_8 (e^-)	$H_2V_4O_8$ (e^-)	$H_4V_4O_8$ (e^-)
V1	0.0258	-0.0227	-0.0557
V2	0.0256	-0.0215	-0.2668
V3	-0.0176	-0.079	-0.2832
V4	-0.0178	-0.079	-0.0677
O1	-0.0817	-0.0968	-0.3701
O2	-0.0712	-0.0959	-0.3553
O3	-0.0267	-0.0987	-0.1493
O4	-0.0385	-0.0983	-0.1842
O5	-0.0796	-0.2649	-0.3588
O6	-0.2707	-0.2737	-0.3185
O7	-0.0597	-0.0833	-0.1321
O8	-0.0581	-0.0829	-0.1657
H1	***0.6703***	***0.6522***	***0.6756***
H2	--	***0.6446***	***0.6885***
H3	--	--	***0.6771***
H4	--	--	***0.6663***
